# Coping with COVID-19 Pandemic and Sustained Health Behavior: A Cross-Sectional Study in Bangladesh

**DOI:** 10.3390/epidemiologia4010009

**Published:** 2023-03-20

**Authors:** Monaemul Islam Sizear, Gloria Macassa, Mohammad Rocky Khan Chowdhury, Mamunur Rashid

**Affiliations:** 1 Open Development LLC, and Public Health Foundation of Bangladesh, 54, Inner Circular Road, Dhaka 1000, Bangladesh; 2 Department of Public Health and Sports Science, Faculty of Health and Occupational Studies, University of Gävle, Kungsbacksvägen 47, 80176 Gävle, Sweden; 3 Department of Epidemiology and Preventive Medicine, School of Public Health and Preventive Medicine, Faculty of Medicine, Nursing and Health Sciences, Monash University, Melbourne 3800, Australia

**Keywords:** coping, COVID-19 pandemic, health behavior, sustainable, stress

## Abstract

During the COVID-19 pandemic, people’s health behavioral changes have been transposed into a new dimension. Coping with the COVID-19 pandemic may have an impact on sustained health behavior (SHB). Therefore, this study aimed to explore the validity and reliability of the COVID-19 Coping Scale among working-age individuals and to assess whether coping with COVID-19-related stress could influence SHB in this population. A cross-sectional study was conducted based on the population of the city of Dhaka in Bangladesh. A total of 263 working-age individuals (19–65 years) participated in the study. The present study results confirmed the COVID-19 Coping Scale was a valid and reliable instrument for this population. Moreover, the present finding indicated decreased odds of SHB for individuals who rated lower scores on coping with COVID-19 compared to individuals who rated higher scores; the result remained significant after controlling for gender and education (OR 0.68, 95% CI: 0.54–0.87). The present study suggests two important findings: (i) the instrument used in this study was valid and reliable in this population, and (ii) coping with COVID-19-related stress may be an important aspect of practicing SHB. Policymakers may use the highlighted findings to facilitate sustainable health behavior for long-term health benefits and to tackle future pandemics like COVID-19 or in a similar context.

## 1. Introduction

Sustained health behavior (SHB) is one of the key health-promoting behaviors to prevent pandemics and to improve population health in general that may contribute to achieving Sustainable Development Goals (SDGs) [1,2,3]. Sustainable development has been a leading and highly acknowledged issue for the United Nations since 2015, and the World Health Organization (WHO) declared that health is the prime aspect of achieving SDGs [4]. There is no consensus on the definition of “SHB” and a paucity of literature has also been observed in this regard. Additionally, practical strategies and theoretical aspects are yet to be well-identified to understand SHB [5]. However, it has been evident that SHB is a process of behavioral change through an intention of sustaining health behavior, by considering individuals’ physical, psychological, and social aspects, toward sustainable health and quality of life [3,6]. SHB has received much attention in the last few years, predominantly during the COVID-19 pandemic [3]. This mostly indicates frequent handwashing, physical distancing, and wearing of masks [7] that could be used to control the spreading of COVID-19 and mitigate its negative impact on population health [8].

The COVID-19 pandemic is considered an urgent global health crisis that greatly affects people’s physical and psychological well-being [9,10]. The rapid spreading of the pandemic worldwide makes people fear, worry, and become distressed, and negatively affects people’s daily quality of life [11,12]. Dealing with the pandemic in such a situation, following strict policies and restrictions such as lockdowns and quarantine, could adversely affect people’s mental health [13]. A cross-sectional study conducted in five European countries (France, Italy, Poland, Sweden, and the UK) found that working from home due to COVID-19 to maintain social distance negatively influenced employed women’s mental health [14]. In Bangladesh, the first COVID-19 case was confirmed on 8 March 2020, and the government followed the lockdown policies to prevent rapid transmission among people from 26 March 2020—this had been extended several times up to the first quarter of 2021. Such policies increased the likelihood of mental health problems among people, particularly those who were living in urban areas [11].

Considering the overall adverse effects brought by the COVID-19 pandemic, coping strategies could play an important role in managing people’s survival as well as strategies for resilience to overcome hardship [15,16]. A coping strategy might remedy negative emotions and stressful events [13]. Similarly, coping with pandemic adversity through employing a specific behavioral and cognitive effort could prevent or lessen stressful events and overcome hardship [17]. The COVID-19 pandemic is a stressful circumstance that affects people’s lives and how people cope with the situation needs to be investigated. A study suggested that positive coping, e.g., maintaining emotion and avoiding stress, indicates positive health status but negative coping indicates negative attitudes towards health problems such as poor physical and psychological conditions [18]. Moreover, the WHO emphasized positive coping mechanisms as an effective approach to dealing with mental health problems under stressful events [19]. A recent study conducted in Vietnam found that emotional support from family and friends is an important aspect of positive coping to alleviate psychological concerns such as stress, anxiety, and depression, particularly in young adults [20].

Little is known yet about whether coping strategies related to the COVID-19 pandemic may have been linked to SHB. Being accustomed to SHB, one’s health behavior may eventually be transformed into a sustainable health behavior [8]. Considering the importance of sustainable health behavior, it is essential to understand how a coping strategy under the COVID-19 pandemic contributes to practicing SHB, particularly among urban–educated people. Due to better access to media and/or social media, the preventive awareness message of COVID-19 was used to spread knowledge in order to educate people on how to deal with the pandemic [21]. Thus, the study focused on assessing whether coping with COVID-19 could influence SHB among the targeted educated people. Previous evidence indicates that people are more likely to suffer negative psychological and trauma-related events during stressful events such as the COVID-19 pandemic, especially when they do not follow any coping strategy [22]. To the best of the authors’ knowledge, no study has investigated how coping strategies, i.e., positive or negative, may have an impact on SHB, especially in the context of Bangladesh. Further, SHB change is needed to reduce the risk of infectious diseases and to prevent and tackle future pandemics [3,6]. According to the information we received, the COVID-19 Coping Scale was used first time in the present study, although the scale was used for clinical purposes. Therefore, the present study first aimed at exploring the validity and reliability of the instrument among working-age individuals (18–65 years) and second, assessing whether coping with COVID-19-related stress could influence SHB in this population.

## 2. Materials and Methods

### 2.1. Study Design and Sampling

A cross-sectional study was conducted based on an online survey among the population of the city of Dhaka in Bangladesh. The targeted population was based on the following criteria: (i) individuals who lived in Dhaka, (ii) working-age adults aged between 18 and 65 years, focusing on the educated cohort (minimum secondary/higher-secondary level), (iii) individuals who were able to use smartphones and/or laptops. The reason behind selecting this group was because the group was susceptible to COVID-19 during the pandemic as most of them were working and used to going outside. They were also convenient to reach through various online platforms during the peak of COVID-19.

The sample size was calculated considering a 5% error rate, 95% confidence interval, and standard deviation of coping strategies amid COVID-19 based on a previous study [16]. To achieve a power of 80%, a minimum of 175 individuals were required for this study, and a total of 263 individuals responded to the survey and took part in the present study.

### 2.2. Data Collection

A combination of purposive and snowball sampling techniques was used for collecting data in this study. Purposive sampling was used to reach a particular group of people following the inclusion criteria. The snowball sampling technique supported us in recruiting the required number of appropriate samples. An online survey based on a self-administered questionnaire created in Google Forms was used to collect data from the participants. The data collection was conducted from 28 July to 27 August 2021 during the peak time of COVID-19 in the capital city of Dhaka, Bangladesh. The online survey progressed upon confirming respondents’ volunteered participation. The survey invitation was primarily sent on social media platforms, and a personal channel via WhatsApp, regular phone messages, and e-mail were also used where a snowball sampling technique was followed to reach out to the potential respondents.

The online survey contained a brief background of the study’s purpose and a consent form to acquire consent from the participants who wanted to take part in the study. The online survey questionnaire was divided into three parts: first, socio-demographic variables (i.e., age, gender, and education); health-seeking behavior during the COVID-19 pandemic was included in second part; and the third part was formulated by an instrument—the COVID-19 Coping Scale (i.e., how respondents dealt with stressful situations in the underlying conditions during COVID-19 pandemic).

### 2.3. Measures

#### 2.3.1. Sustained Health Behavior

The outcome variable of the study was SHB (i.e., an intention of sustaining health behavior) dichotomized as “yes” or “no”. Three guidelines from the WHO—handwashing, maintaining physical distance, and wearing a mask—were considered for measuring SHB. To measure SHB, the following question was asked: “Do you want to continue the habits of handwashing, wearing masks, and maintaining physical distance even when the COVID-19 pandemic will start to fade away?” The participants who had an intention of maintaining SHB were categorized as “yes”; otherwise, they were categorized as “no”.

#### 2.3.2. Coping with COVID-19-Related Stress

In the present study, the COVID-19 Coping Scale, developed by an American clinical psychologist, was used for measuring coping with COVID-19-related stress [23]. The original scale consists of 15 items (7 represent positive coping and 8 indicate negative coping), where responses are made on a 5-point Likert rating scale (0 = not at all, 1 = a little, 2 = moderately, 3 = quite a bit, and 4 = extremely).

#### 2.3.3. Covariates

Information about COVID-19 status was collected by asking a question about whether the participants had COVID-19 or not during the pandemic period. Information about handwashing, physical distancing, and wearing masks was also recorded as a dichotomous variable (yes or no). In addition, demographic variables such as age, gender, and education were collected through background questions that were used as covariates in the analysis.

#### 2.3.4. Data Analysis

Prior to performing the analyses, the normality of the data was checked using scatterplots. No outliers in the data were observed. Descriptive statistics were presented as proportions, means, and standard deviations.

To assess the factorial validity and reliability of the instrument, the following steps were taken. First, principal factor analysis was performed using Kaiser–Meyer–Olkin (KMO) to measure of the adequacy of sampling, and the Bartlett test of sphericity to see whether the data structure was appropriate for factor analysis. Second, the factor that consisted of similar items became two sub-scales, i.e., positive and negative coping. An index was made by calculating an average positive and negative coping value separately, represented as 0 to 4 and 0 to −4, respectively. Afterward, these two sub-scales were merged into one scale called coping with COVID-19 where a higher positive score indicates positive coping, and a higher negative score indicates negative coping, i.e., a distressed condition. Third, a reliability test using Cronbach α for the scale and sub-scales was calculated.

The difference in prevalence of SHB by background characteristics was evaluated with an independent *t*-test for age and coping with COVID-19; a chi-square test for gender, physical distancing, and COVID-19 status; Fisher exact test for education, handwashing, and wearing a mask. Finally, a multivariate logistic regression model was executed to see whether coping with COVID-19 has an impact on SHB. Nagelkerke’s pseudo-R^2^ was used to measure the goodness-of-fit of the model. The level of significance was set at *p* < 0.05. The statistical program IBM SPSS version 27 was used for the analyses.

## 3. Results

The mean age of the study participants was 35 years, standard deviation was 9.0, and approximately 58% were male. More than 80% had graduate or postgraduate education. Around 36% of the participants tested COVID-19 positive. Out of 263 individuals, 49% wanted to continue with the SHB, whereas 51% did not (Table 1).

Principal factor analysis was performed using KMO to measure the adequacy of sampling and the KMO result was 0.83, which indicates a high level of sampling adequacy. The Bartlett test of sphericity result was significant (*p* < 0.001), indicating that the data structure is appropriate for factor analysis. Table 2 indicates the factor loading of 12 items where factor 1 (positive coping) loads values ranging from 0.60 to 0.76, and factor 2 (negative coping) constructs values tightly ranging from −0.17 to 0.18.

The internal consistency of the scale and sub-scales using Cronbach alpha coefficients ranged from 0.78 to 0.84, indicating high consistency (Table 3).

Table 4 represents a bivariate analysis of the participants’ background characteristics and coping with COVID-19 between groups—those who did continue with SHB and those who did not continue with SHB. The variables—gender, education, and negative coping—differ significantly between the groups.

Table 5 indicates decreased odds of SHB for individuals who had lower scores on coping with COVID-19 compared to individuals with higher scores; the result remained significant after adjusting for gender and education (OR 0.68, 95% CI: 0.54–0.87). Nagelkerke’s pseudo-R^2^ of the adjusted model was 22%, and the model was significant at *p* < 0.001.

## 4. Discussion

This cross-sectional study was undertaken to explore the validity and reliability of the scale among working-age individuals (18–65 years) living in an urban area in Bangladesh and to assess whether coping with COVID-19-related stress could influence SHB in this population. The instrument (scale and sub-scales) in the present study was found to be valid and reliable. The study also indicated that individuals who negatively coped with COVID-19-related stress were less likely to be associated with SHB, compared to individuals who positively dealt with stress.

As mentioned earlier, to the best of the authors’ knowledge, no study has explored the validity and reliability of the COVID-19 Coping Scale using a population-based sample. Data obtained from Dhaka, Bangladesh confirmed the two-factor structure, i.e., positive and negative coping. The COVID-19 Coping Scale is a valid and reliable instrument for this population. The result is consistent with a similar study focused on the same subject, but in a different socio-economic setting [24].

The present finding regarding coping with COVID-19-related stress and SHB is in agreement with a previous study, although the outcome of that study was not directly related to SHB, indicating that effective coping strategies during a crisis such as the COVID-19 pandemic play a significant role in managing distress [15]. Coping with stress conditions for a certain period may change one’s health behavior [25]. One possible explanation for this result could be that individuals in the present study who answered “yes” to SHB had an increased coping strategy to deal with COVID-19-related stress. Moreover, individuals who have self-control tend to follow suggested health behavior such as handwashing, maintaining a physical distance, and wearing masks. A previous study also found that people follow better coping strategies when they have more self-control [26].

Education may play a pivotal role in controlling one’s behavioral change to avoid negative consequences and improve positive outcomes. Education, as a confounding factor in this study, was found to be significant in the adjusted analysis. A previous study also found education as an important factor that may contribute to maintaining a positive coping mechanism [27]. One of the possible reasons could be that educated people are more likely to cope with any distressing situation by controlling their behavior and tend to follow suggested behavioral guidelines on the emerging situation. An earlier review regarding health behavioral aspects concluded that sustained behavioral changes among people require long interventions by developing knowledge of them [5].

### 4.1. Strengths and Limitations

The main strength of the present study is the aim to understand the SHB aspect when it comes to coping with COVID-19-related stress, which has not yet been widely explored. Another strength is that the COVID-19 Coping Scale used in this study was not employed in any empirical research previously, and the instrument was shown to have adequate internal validity and reliability in this population.

Despite the potential implications of the present study, the study still has some limitations that should be noted. First, the survey was only based in Dhaka, the capital of Bangladesh, and the sample size was small. In addition, the data were collected from a particular group; that is, the participants who took part in the survey were urban dwellers and they had a mostly high level of education. Due to this reason, the findings from the present study cannot be generalized to individuals from rural areas and individuals with no education in a similar context. Moreover, this study did not perform stratified analysis by gender; thereby, it should not be asserted whether gender plays any role in this study. Further, gender was considered a confounder in the adjusted analysis, which did not reveal a significant result concerning SHB. Further research is warranted covering a broad range of socio-economic backgrounds of individuals, including urban and rural, and considering gender differences, which would provide a broader picture and give a better understanding in this regard. Likewise, a qualitative study design could be useful to be able to gain a deeper understanding of individuals’ coping with stress and SHB.

### 4.2. Implications of the Study Findings

Considering the major findings of the study, this study may be considered by policymakers and public health practitioners to facilitate SHB for long-term health benefits by promoting how to deal with any stressful event. In addition, the result of this study may potentially be useful for an effective intervention on SHB addressing individuals’ psychological health to tackle future pandemics or in a similar context.

The validity and reliability of the instrument—the COVID-19 Coping Scale—could contribute to the empirical literature dealing with coping and pertaining to pandemic-related stress. Notably, this study found 12 items out of 15 that fairly formulated two sub-scales—positive and negative coping—that would guide a new direction for future research in this regard. Although the present results confirmed content validity, convergent validity, factor validity, and appropriate reliability, further study is needed in different settings before recommending the instrument for other populations.

## 5. Conclusions

The present study confirmed that the COVID-19 Coping Scale was a valid and reliable instrument for this population. The present finding also suggested that coping with COVID-19-related stress may be an important aspect of practicing SHB. Policymakers and public health practitioners may use the highlighted finding to facilitate SHB for long-term health benefits, and even to prevent or tackle future pandemics like COVID-19.

## Figures and Tables

**Table 1:** Background characteristics of the study population (*n* = 263).

Characteristics	Frequency (%)
Age (M ± SD)	35 ± 9.0
Gender	
Male	154 (58.6)
Female	109 (41.4)
Education level	
Secondary/higher secondary	39 (14.9)
Undergraduate/Graduate	214 (81.1)
Postgraduate	9 (3.4)
Handwashing	
Yes	252 (96.2)
No	10 (3.8)
Wearing mask	
Yes	240 (91.3)
No	23 (8.7)
Physical distancing	
Yes	103 (39.2)
No	160 (60.8)
COVID-19 status	
Yes	74 (28.1)
No	127 (48.3)
Missing	62 (23.7)
Sustained health behavior	
Yes	129 (49.0)
No	134 (51.0)

M—Mean, SD—Standard Deviation.

**Table 2:** Principal component analysis—factor loadings.

Items	Factor	
	1	2
Can focus	0.76	0.18
Feel supported at work	0.75	0.01
Can remember things	0.71	0.04
Can enjoy things	0.68	0.20
Feel calm	0.61	0.19
Feel like coping	0.60	−0.17
Feel tired	0.14	0.78
Feel worried	−0.05	0.77
Worried about mental health	0.19	0.77
Feel uncertain	−0.06	0.74
Feel angry	0.05	0.72
Feel confused	0.19	0.71

Factor 1 (Positive coping): Can focus + Feel supported at work + Can remember things + Can enjoy things + Feel calm + Feel like coping; Factor 2 (Negative coping): Feel tired + Feel worried + Worried about mental health + Feel uncertain + Feel angry + Feel confused.

**Table 3:** Reliability of the scale and sub-scales.

Scale and Sub-Scales	*n*	Mean	SD	Cronbach α
Coping with COVID-19	256	−0.25	1.07	0.81
Positive coping with COVID-19	254	10.97	4.52	0.78
Negative coping with COVID-19	258	−12.36	5.83	0.84

**Table 4:** Characteristics and coping with COVID-19 in terms of sustained health behavior among working-age people aged 19–65 years (*n* = 263).

Background Variables	Sustained Health Behavior		*p*-Value
	Yes	No	
Age, years (M ± SD)	34.65 ± 7.9	35.60 ± 10.0	0.39
Gender, *n* (%)			0.03
Male	84 (65.1)	70 (52.2)	
Female	45 (34.9)	64 (47.8)	
Education level, *n* (%)			0.002
Secondary/higher secondary	9 (7.0)	30 (22.6)	
Undergraduate/graduate	113 (87.6)	101 (75.9)	
Postgraduate	7 (5.4)	2 (1.5)	
COVID-19 status, *n* (%)			0.85
Yes	60 (62.5)	67 (63.8)	
No	36 (37.5)	38 (36.2)	
Positive coping (M ± SD)	1.82 ± 0.7	1.87 ± 0.7	0.58
Negative coping (M ± SD)	−2.10 ± 0.8	−1.86 ± 0.9	0.02
Coping with COVID-19 (M ± SD)	−0.31 ± 1.1	−0.01 ± 1.2	0.04

The range of the positive and negative coping scale was (0 to 4) and (0 to −4), respectively; M, Mean; SD, Standard Deviation.

**Table 5:** Association between coping with COVID-19 pandemic and sustained health behavior among working-age people aged 19–65 years.

Variable	Unadjusted Analysis			Adjusted Analysis		
	SE	OR (95% CI)	*p*-Value	SE	OR (95% CI)	*p*-Value
Coping with COVID-19	0.11	0.80 (0.65–0.98)	0.04	0.12	0.68 (0.54–0.87)	0.002
Gender				0.30	0.65 (0.36–1.17)	0.15
Education				0.33	2.68 (1.40–5.11)	0.003
Overall model	R^2^ = 0.03, χ^2^ = 4.45, *p* = 0.03			R^2^ = 0.22, χ^2^ = 24.57, *p* < 0.001		

## Data Availability

The dataset is available from the corresponding author upon reasonable request.
